# Disturbances in the Glutathione/Ophthalmate Redox Buffer System in the Woodchuck Model of Hepatitis Virus-Induced Hepatocellular Carcinoma

**DOI:** 10.1155/2011/789323

**Published:** 2011-09-18

**Authors:** Rafael Andres Ibarra, R. Abbas, R. S. Kombu, Guo-Fang Zhang, G. Jacobs, Z. Lee, H. Brunengraber, J. R. Sanabria

**Affiliations:** ^1^Department of Surgery, Case Western Reserve University School of Medicine and University Hospitals, Case Medical Center, Cleveland, OH 44106-7029, USA; ^2^Department of Nutrition, Case Western Reserve University School of Medicine and University Hospitals, Case Medical Center, Cleveland, OH 44106-7029, USA; ^3^Department of Pathology, Case Western Reserve University School of Medicine and University Hospitals, Case Medical Center, Cleveland, OH 44106-7029, USA; ^4^Department of Radiology, Case Western Reserve University School of Medicine and University Hospitals, Case Medical Center, Cleveland, OH 44106-7029, USA; ^5^Division of Transplant and Hepatobiliary Surgery, Department of Surgery, University Hospitals, Case Medical Center, Lakeside 7510, PS 5047, Cleveland, OH 44106, USA

## Abstract

*Purpose*. The incidence of liver tumors is rising in USA. The purpose of this study was to evaluate liver oxido-reductive status in the presence of chronic liver disease and hepatocellular carcinoma (HCC). *Methods*. Glutathione species and ophthalmate (OA) concentrations were measured by LC-MS in processed plasma and red blood cells (RBC) from infected Woodchuck with hepatitis virus (WHV). Blood samples were obtained from: (i) infected animals with tumors (WHV+/HCC+), (ii) infected animals without tumors (WHV+/HCC−) and (iii) healthy animals (WHC−/HCC−). *Results*. The concentration of reduced glutathione (GSH) and the ratio GSH/GSG were lower in plasma from WHV+/HCC+ animals when compared to WHV+/HCC− and WHV−/HCC− (*P* < 0.01). In contrast, the concentration of oxidized glutathione (GSSG) was found to be higher in plasma from WHV+/HCC+ animals when compared to WHV+/HCC− and WHV−/HCC− (*P* < 0.01). The Glutathione species and its ratio from the RBC compartment were similar among all groups. OA concentration in both plasma and RBC was significantly higher from WHV+/HCC+ when compared to WHV+/HCC− and WHV−/HCC− (*P* < 0.01). *Conclusions*. Disturbances of the glutathione redox buffer system and higher concentrations of OA were found in the WCV+/HCC+ animal model. The role of these compounds as biomarkers of early tumor development in patients with end stage liver disease remains to be determined.

## 1. Introduction

Hepatocellular carcinoma (HCC) is the fourth most common cancer in the world, with a high lethality index [1]. It develops and flourishes mostly in patients with liver disease, and its incidence is on the rise in United States due to an increase in hepatitis C virus (HCV) infection and nonalcoholic steatohepatitis (NASH) [1]. 80% of these tumors are surgically untreated at the time of diagnosis because of an advanced stage or due to a medical condition that prohibits resection or transplantation, the only curative treatments [2]. Screening programs in high-risk populations including serial *α*-fetoprotein measurements and liver ultrasonography had failed their aim towards early tumor detection [3–5]. Authors have postulated metabolic substrates as a form of biomarker for the early detection of liver cancer [6].

The reduced glutathione : oxidized glutathione (GSH : GSSG) couple is the most important intracellular buffer system against oxidative stress [7, 8]. Perturbations of this system and other oxidative stress changes have been previously reported in relation to different cancers [7–9]. Soga et al. in 2006 showed that serum levels of ophthalmate (OA), an analog of glutathione, can be used as an index of intracellular GSH depletion/oxidative stress [10]. We have shown that disturbances of the glutathione redox buffer system and OA were present in the VX2 rabbit model of secondary liver tumors as early as 14 days after tumor implantation and these changes were caused by an oxidative stress reaction within the tumor surroundings in response to the neoplastic growth [11]. In the present work, we hypothesized that these changes can also be detected in animals with chronic viral hepatitis-induced HCC and that complete ablation of the tumors will not altered the disturbances of the glutathione/OA buffer system observed in early tumor development, since these are produced in the surrounding healthy liver tissues in reaction to a growing mass in need for energy substrates. To answer this question we made use of the well-established woodchuck hepatitis virus- (WHV-) induced HCC model [12, 13]. Summers et al. in 1978 reported a high incidence of HCC (23%) arising in a background of chronic active hepatitis in a colony of captive woodchucks studied for 18 years [14]. Gerin et al. in 1989 described that almost 97% of chronic WHV carriers developed HCC within 3 years of WHV experimental inoculation [15]. We found a significant decrease in the GSH/GSSG ratio and an increase in the concentrations of OA in plasma from infected animals with tumors when compared to both infected animals without tumors and healthy controls (noninfected animals without tumors).

## 2. Materials and Methods

### 2.1. Animal Model

Woodchucks *(marmota monax)*, 21–36 months old, weighing 2.5–2.75 kg (Northeastern Wildlife Inc., Harrison, Idaho, USA) were quarantined for 15 days and maintained in fixed conditions of day-light cycle and humidity with access to water and food (Teklad, Madison, Wis, USA) *ad libitum*. They were fasted overnight before any procedure. All animal protocols were approved by the Institutional Animal Care and Use Committee (IACUC) at Case Western Reserve University and conducted in accordance to their guidelines. Animal groups consisted of: (i) *healthy *woodchucks (noninfected without tumors = WHV−/HCC−, *n* = 4), (ii) chronic WHV-*infected *woodchucks *without tumors* (WHV+/HCC−, *n* = 4), and (iii) *experimental* animals (WHV-infected with tumors = WHV+/HCC+, *n* = 4 − 1). One male animal from the experimental group became ill during the quarantine period and was excluded from the protocol.

### 2.2. Tumor Ablation

WHV+/HCC+ animals were anesthetized with intramuscular (IM) Xylazine (5 mg/kg) and Ketamine (50 mg/kg). IV fluids were administered through a sublingual vein catheter and their abdominal wall was shaved and swabbed with povidone-iodine solution (Betadine, Mich, USA) prior to a midline laparotomy. Livers were inspected and tumors were ablated with a Valleylab Cool-tip RF ablation system (Valleylab/Tyco Healthcare Group, Boulder, Colo, USA) for 12 minutes under continuous impedance. Ablation electrodes included a 0.7 cm probe for a 1.5 cm ablation area and a 1 cm probe for a 2 cm ablation area. The size of the ablation electrode was selected in a case-by-case basis according to the tumor size. A total of 2 to 3 ablations were performed per animal with the goal of complete tumor ablation.

### 2.3. Blood Sampling

Baseline blood samples were collected from all groups in a heparinized tube before any procedure and repeated 8 hours after tumor ablation in the experimental group. Samples were immediately cooled down in iced water. After centrifugation at 3,000 rpm at 4°C for 10 min, the plasma and red blood cells (RBCs) were separated in cryogenic vials for GSH/GSSG/OA assay. To prevent its oxidation, GSH was immediately converted to a stable carboxymethyl thioether by treating 500 *μ*L of plasma with 500 *μ*L of 50 mM iodoacetate in 10 mM ammonium bicarbonate, pH = 10, adjusted with concentrated ammonia. After removal of the plasma, 50 *μ*L of the RBC pellet was transferred to a cryogenic vial and treated with 450 *μ*L of IAA buffer [16]. Snap-frozen aliquots of plasma and RBC were stored at −80°C until analysis.

### 2.4. Imaging Procedures

In order to demonstrate the presence of liver tumors, we obtained positron emission tomography (PET) scans 1 week before surgery with three different tracers: (i) 2-deoxy-2[^18^F]-fluoro-D-glucose (*FDG*), (ii) [1-^11^C]-acetate (*acetate*), and (iii) [N-methyl-^11^C]-choline (*choline*). Tracers were synthesized in our lab [17]. Two postoperative PET scans were performed at 4 and 7 hours after tumor ablation with acetate (15 minutes after giving the radioisotope) and FDG (1 hour after giving the radioisotope), respectively. The Allegro PET scanner (Philips Medical Systems Inc., Cleveland, Ohio) was used with three-dimensional acquisition. Images were reconstructed using filtered backprojection with attenuation correction using the transmission scan [17].

### 2.5. Pathologic Analysis

Eight hours after RFA of the tumors, woodchucks were euthanized and their livers removed. Liver were perfused-fixed with 10% formaldehyde and 90% phosphate-buffered saline at room temperature. Liver tissue was embedded in paraffin blocks to be cut and then Hematoxylin and Eosin stained. Blinded slides were assessed by two pathologists specialized in liver disease. Digital records from tumors were used to determine their size (Digi 3 Digital Binocular Microscope and software, LaboMed, Calif, USA).

### 2.6. GSH/GSSG/OA Assay

Plasma and RBC concentrations of glutathione species GSH : GSSG and OA were measured using LC-MS/MS methods validated in our laboratory [16]. In brief, the protected plasma samples were spiked with homoglutathione as internal standard (Chem-Impex International Inc., Wood Dale, Ill, USA). For the RBC samples, 10 *μ*L of the protected sample was diluted in 90 *μ*L of water and spiked with the same amount of homoglutathione. Iodoacetate treated samples were kept in the dark at room temperature for 45 min to allow completion of the reaction. To liberate the bound glutathione, 200 *μ*L of DTT (100 mM, pH 10, in 10 mM ammonium bicarbonate) was added and allowed to react in the dark for 15 min at room temperature. To this solution, 200 *μ*L of iodoacetonitrile (200 mM, pH 10, in 10 mM ammonium bicarbonate containing 3.12 *μ*M homoglutathione (623 pmol/200 *μ*L)) was added to convert the liberated glutathione to a cyanomethyl-thioether. After 30 min standing at room temperature in the dark, 1.5 mL of acetonitrile was added to precipitate the proteins. After completing all reactions, sample was dried at 50°C under air at 20 psi for 40 min and reconstituted in formic acid in water (0.1% vol : vol). This was injected into a liquid chromatograph (Agilent 1100, Agilent Technologies Inc., Palo Alto, CA) equipped with an API 4000 QTrap mass spectrometer (Applied Biosystems, Foster City, CA) operated under positive ionization mode. A Hypersil Gold C18 column (2.1 × 150 mm, 5 *μ*m particle size; Thermo Electron Corp.) was used at room temperature. Mobile phase A was 0.15% formic acid in water-acetonitrile (99 : 1, vol : vol), and mobile phase B was 0.15% formic acid in water-acetonitrile (5 : 95 vol : vol). Using a gradient elution, the compounds were eluted at a flow rate of 0.2 mL/min. Analyst software (version 1.4.1; Applied Biosystems, Foster City, Calif) was used for data registration and analysis. The MRM ion pairs monitored (precursor → product) were (i) for carboxymethyl-GSH: 366.1 → 237.2, (ii) for cyanomethyl-GSH derived from GSSR: 347.2 → 272.1, (iii) for carboxymethyl-homoglutathione: 380.1 → 233.1, (iv) for cyanomethyl-homoglutathione: 361.1 → 232.1, and (v) for ophthalmate: 290.3 → 161.1. The GSH/GSSG ratio was calculated using the formula (GSH)/(GSSG/2).

### 2.7. Statistical Analysis

Mean values ± standard deviations are presented (M ± SD). One-sided paired *t*-tests were used to calculate significance of difference in the mean values from same animals before and after tumor ablation. One-sided independent *t*-tests were used to calculate significance of difference in the mean values between the experimental group and other groups. A *P* value <0.05 was considered statistically significant.

## 3. Results

**Figure 1:**
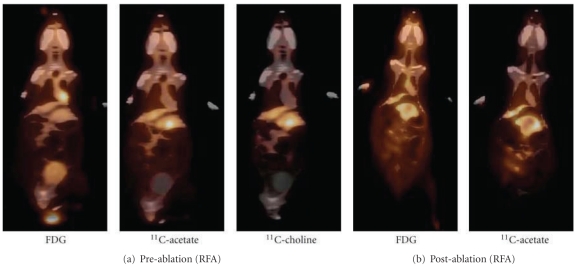
Radioactive tracer uptake of livers from WHV-induced HCC woodchuck model using ^18^F-glucose (FDG), ^11^C-acetate and ^11^C-choline. (a) Representative imaging of an animal with WHV-induced HCC in which the uptake of FDG was negative while the uptake of ^11^C-acetate and ^11^C-choline were positive. (b) After radiofrequency ablation (RFA) of the tumor the uptake of FDG and ^11^C-acetate were lower than the surrounding liver tissue.

**Figure 2:**
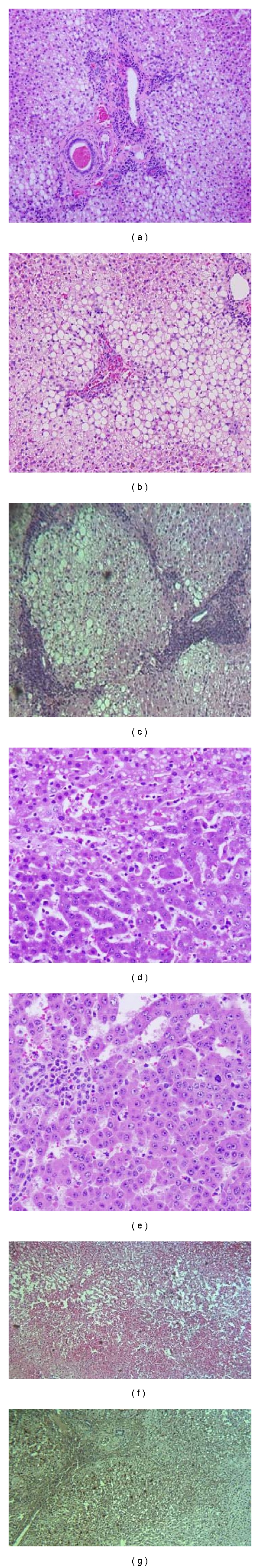
Light microscopy of liver tissue from WHV-infected woodchucks. (a) Liver tissue is shown with areas of mild chronic hepatitis, steatosis, and mild portal fibrosis, characteristic of mild WHV infection (H&E, 100x), while other areas showed portal triad fibrosis with severe steatosis (b) progressing to bridging fibrosis (c). (d) A region of severe dysplasia versus HCC (H&E, 200x). (e) HCC with minimal steatosis containing mononuclear cells. (f) Liver tissue containing malignancy after complete tumor ablation (H&E, 100x) and a 2 mm margin around ablated area showing cell apoptosis (g), (TUNEL assay, 100x).

PET scans 1 week before tumor ablation showed that the uptake of FDG in all tumor bearing woodchucks 1 hour after isotope injection was similar to the surrounding hepatic tissues (tumor-to-liver ratio ≤1.15, Figure 1(a), FDG). In contrast, when using acetate and choline as radiotracers a focal uptake in HCC was evident (Figure 1(a), acetate and choline). Scans after tumor ablation showed a lower uptake of FDG and acetate than the surrounding hepatic tissues in all ablated areas, and no hypermetabolic focus was seen (Figure 1 (b)). No signs of metastasis were noted at imaging or during surgery. Liver tumor masses were evident in all experimental animals during surgery. The mean diameter of tumors was 4.35 ± 1.75 mm. Histologic examination showed mild-to-moderate chronic hepatitis, mild-to-moderate steatosis and mild portal fibrosis progressing to bridging fibrosis. Conclusive malignancies were present in two experimental animals while controversy was present between pathologists for the third animal: HCC versus highly dysplastic nodules (Figure 2). Animals in other groups showed no evidence of malignancy.

**Figure 3:**
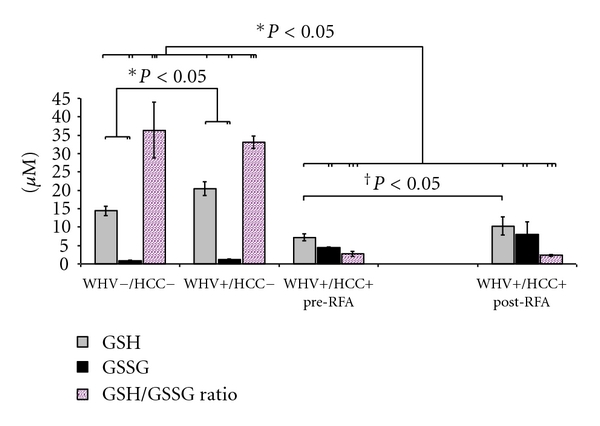
Concentration of glutathione species and their ratio in plasma from the WHV-induced HCC woodchuck model (*μ*M). The reduced form (GSH), the oxidized form (GSSG) of glutathione and their ratio were measured in processed plasma samples of healthy woodchucks (WHV−/HCC−), WHV-infected woodchucks (WHV+/HCC−), and WHV-induced HCC animals (WHV+/HCC+, before and 8 hours after tumor ablation). Levels of GSH were lower in the WHV+/HCC+ group when compared to the WHV−/HCC− and WHV+/HCC− groups. In contrast, levels of GSSG were higher in WHV+/HCC+ when compared to WHV−/HCC− and WHV+/HCC−. The GSH/GSSG ratio presented a ~12-fold decrease in plasma from WHV+/HCC+ animals when compared to controls. Levels of GSH increased significantly after tumor ablation in the WHV+/HCC+ animals. *P < 0.05, one-sided independent *t*-test; ^†^ *P* < 0.05, one-sided paired *t*-test.

**Figure 4:**
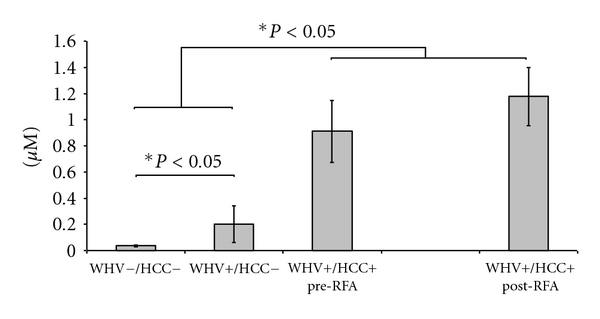
Concentration of ophthalmate (OA) in plasma from the WHV-induced HCC woodchuck model (*μ*M). The concentrations of OA were measured in plasma samples of healthy woodchucks (WHV−/HCC−), WHV-infected woodchucks (WHV+/HCC−) and WHV-induced HCC animals (WHV+/HCC+, before and 8 hours after tumor ablation). Levels of OA were significantly higher in the WHV+/HCC+ when compared to WHV+/HCC− and WHV−/HCC−. OA levels were also higher in WHV+/HCC− when compared with the WHV−/HCC−. Levels of OA were similar in the WHV+/HCC+ before and after tumor ablation. *P < 0.05, one-sided independent *t*-test.

*Plasma* concentration of GSH was significantly lower in the WHV+/HCC+ group when compared to WHV+/HCC− and to WHV−/HCC− groups (7.23 ± 0.95 versus 20.50 ± 1.90 versus 14.44 ± 1.25 *μ*M, *P* = 0.0002, Figure 3). In contrast, the concentration of GSSG was significantly higher in the WHV+/HCC+ when compared to WHV+/HCC− and to WHV−/HCC− (4.45 ± 0.13 versus 1.25 ± 0.17 and 0.83 ± 0.25 *μ*M, *P* = 0.003, Figure 3). The GSH/GSSG ratio was 12.1 to 13.4 times lower in the WHV+/HCC+ animals when compared to the WHV+/HCC− and to WHV−/HCC− groups, respectively (2.71 ± 0.68 versus 33.06 ± 1.72 and 36.38 ± 7.55, *P* = 0.0003, Figure 3). The plasma concentration of ophthalmate was increased 4.5-fold in the WHV+/HCC+ group when compared to the WHV+/HCC− animals and 22-fold when compared to the WHV−/HCC− (0.91 ± 0.24 versus 0.20 ± 0.14 and 0.04 ± 0.01 *μ*M, *P* = 0.002, Figure 4). Concentrations of GSH from the WHV+/HCC+ woodchucks increased significantly 8 hours after tumor ablation when compared to their baseline levels (7.2 ± 0.9 → 10.3 ± 2.5 *μ*M, *P* = 0.048, paired *t*-test, Figure 3). The changes in concentration of GSSG, OA and the GSH/GSSG ratio before and after tumor ablation were not statistically significant (*P* > 0.05, paired *t*-test).

**Figure 5:**
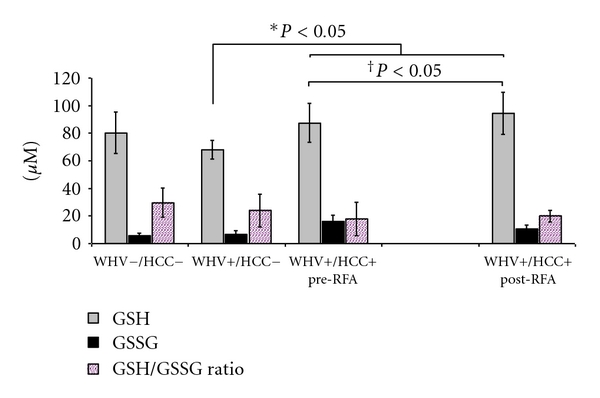
Concentration of glutathione species and their ratio in RBC samples from the WHV-induced HCC woodchuck model (*μ*M). The reduced form (GSH), the oxidized form (GSSG) of glutathione and their ratio were measured in processed RBC samples of healthy woodchucks (WHV−/HCC−), WHV-infected woodchucks (WHV+/HCC−), and WHV-induced HCC animals (WHV+/HCC+, before and 8 hours after tumor ablation). Levels of GSH were higher in WHV+/HCC+ animals when compared to WHV+/HCC− and it increased significantly after tumor ablation. GSH/GSSG ratio was similar among all groups. *P < 0.05, one-sided independent *t*-test; ^†^ *P* < 0.05, one-sided paired *t*-test.

**Figure 6:**
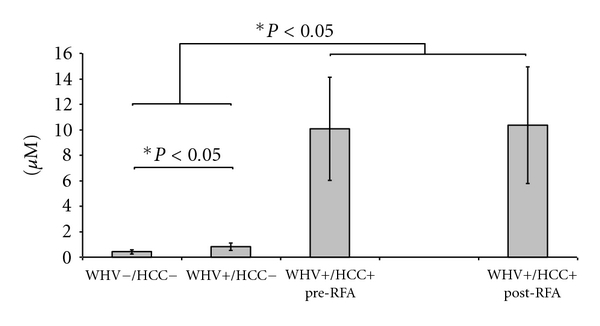
Concentration of ophthalmate (OA) in RBC samples from the WHV-induced HCC woodchuck model (*μ*M). The concentrations of OA were measured in RBC samples of healthy woodchucks (WHV−/HCC−), WHV-infected woodchucks (WHV+/HCC−), and WHV-induced HCC animals (WHV+/HCC+, before and 8 hours after tumor ablation). The level of OA was higher in WHV+/HCC+ when compared to WHV+/HCC− and WHV−/HCC−. OA level was also higher in WHV+/HCC− when compared with the WHV−/HCC−. Levels of OA were similar in the WHV+/HCC+ group before and after tumor ablation. *P < 0.05, one-sided independent *t*-test.

*RBCs* GSH concentration was significantly higher in the WHV+/HCC+ group when compared to WHV+/HCC− group (87.48 ± 14.02 versus 67.93 ± 6.64 *μ*M, *P* = 0.027, Figure 5) and similar to the WHV−/HCC− group (80.30 ± 15.11 *μ*M, *P* = 0.274, Figure 5). RBCs GSSG concentration was similar in WHV+/HCC+ when compared to WHV+/HCC− and to WHV−/HCC− (16.06 ± 4.61 versus 6.61 ± 2.67 and 5.81 ± 2.01 *μ*M, *P* > 0.05, Figure 5). The GSH/GSSG ratio was similar among groups (*P* > 0.05, Figure 5). In contrast, RBCs OA concentration was significantly higher in WHV+/HCC+ animals when compared to WHV+/HCC− and WHV−/HCC− (10.06 ± 4.04 versus 0.81 ± 0.29 and 0.42 ± 0.17 *μ*M, *P* = 0.002, Figure 6). Moreover, the RBCs GSH concentration significantly increased in the WHV+/HCC+ group after tumor ablation when compared to preablation levels (87.48 ± 14.02 → 94.50 ± 15.36 *μ*M, *P* = 0.048, paired *t*-test, Figure 5). The RBCs concentrations of GSSG, OA and the GSH/GSSG ratio remained unchanged after tumor ablation when compared to baseline levels prior ablation (*P* > 0.05).

## 4. Discussion

Cell transformation to the one with a malignant and uncontrolled growth is associated with oxidative stress [7–9]. Previous studies in our lab have shown disturbances of the glutathione redox buffer system and ophthalmate elevation in the VX2 rabbit model of secondary liver tumors [11]. We hypothesized that these findings will also be present in animals with viral hepatitis-induced HCC. Our data show a significant decrease in the plasma concentration of reduced glutathione and GSH/GSSG ratio in animals with WHV-induced HCC when compared to WHV-infected animals without tumors and healthy controls. In addition, oxidized glutathione and ophthalmate concentrations were found to be significantly higher in the WHV-induced HCC animals when compared to the other groups. These findings indicate that a higher state of oxidative stress was present in this animal model of HCC and can be potentially used as a biomarker for early HCC detection in patients with chronic liver disease.

Patients with advanced malignancies are usually in a catabolic state. Authors have shown that high circulating levels of tumor necrosis factor *α* (TNF*α*) and other cytokines associated with tumor growth promoted low energy intake, increased energy expenditure and negative energy balance [18]. Mantovani et al. found a significant increase in the circulating levels of TNF*α* and IL-6 along with a reduced activity of the enzyme glutathione peroxidase in patients with advanced stage tumors (stage III-IV) [19]. Decreased glutathione peroxidase activity is considered a surrogate marker for oxidative stress, this is likely due to a decrease availability of its substrate, reduced glutathione [18, 19]. In addition, the concentration of reduced glutathione in plasma was found to be significantly lower in patients with advanced stages of lung, breast, colon, and renal cancer [7–9]. We have shown for the first time a significant decrease in the plasma concentrations of GSH in woodchucks with HCC and chronic viral hepatitis when compared to animals with hepatitis but without tumors or to healthy animals. The glutathione redox couple is one of the main cellular defenses against oxidative stress [7–10]. Failure of this system may result in mitochondrial failure and apoptosis of healthy cells enhancing a favorable microenvironment for further malignant growth. Reduced glutathione (GSH) protects the cell against reactive oxygen species (ROS) by becoming oxidized to GS***·***, with further formation of glutathione disulfide (GSSG) by combination of 2 GS*·* radicals [20]. Therefore, GSSG is accumulated during oxidative stress, and it can be reduced to GSH via glutathione reductase or preferentially excreted to the plasma [21]. ROS are involved in the etiology of cancer via DNA-oxidative damage, inducing alterations of transcription factors such as p53 [22], nuclear factor kappa B and activator protein 1 [23]. In addition, glutathione disturbances in cancer cells may play a role in the development of metastatic potential and chemo-resistance. Carretero et al. showed an increase concentration of reduced glutathione during* in vitro* growth of a B16 melanoma cell line. Furthermore, intrasplenic inoculation of high GSH concentration cells in mice showed a significantly high incidence of liver metastasis than low-GSH cells 10 days after injection [24]. Cancer cells with a higher GSH content are more resistant to chemotherapy and radiation therapy, since they are more resistant to oxidative stress [21].

The tripeptide glutathione (*γ*-L-Glutamyl-L-cysteinylglycine) is primarily synthesized in the liver, where it reaches concentrations of ~10 mM, from where it is exported to other organs [21, 25, 26]. The labeling profile of plasma glutathione species is similar to the liver: 2%–3% per minute. Since there is no synthesis of glutathione in plasma, the labeling of plasma GSH : GSSG reflects the hepatic synthesis of glutathione. Therefore, alterations in the liver glutathione system can be directly measured in the plasma compartment [16]. The role of the glutathione redox buffer system in erythrocytes has been extensively studied. GSH synthesized in the RBC protects the cell against oxygen radicals and it is essential to maintain the integrity of the cell membrane. The concentration of RBCs glutathione species is ~10 times higher than plasma. Kombu et al. showed that the RBCs glutathione species labeling pattern is ~7 times slower than liver, which indicates a slow rate of synthesis and/or a slow rate of import from the plasma compartment relative to the larger RBCs pool size [16]. For this reason, their concentrations do not reflect the status of the liver glutathione system. Therefore, when using blood samples for glutathione species measurement with the purpose of investigating liver disturbances, samples should be handled and processed carefully (i.e., avoiding hemolysis) so that their concentrations truly reflects the concentration of the liver-plasma compartment. Glutathione species, their ratio and the concentration of OA, was found to be significantly higher in the plasma compartment of animals virus infected and with tumors when compared to animals virus infected without tumors and to healthy controls. In contrast, glutathione ratio was found to be similar in the RBCs compartment in all animals groups. Furthermore, the reduced fraction of glutathione increased 8 hours after tumor ablation in plasma of WHV+/HCC+ animals in both plasma and RBCs compartments, perhaps representing, at least in part, an increased synthesis of reduced glutathione by liver cells after a thermal injury with subsequent release and increased plasma concentration and perhaps percolation into circulating RBCs. 

Ophthalmate is a nonthiol analog of glutathione. It is produced under low cysteine conditions by the same enzymes of glutathione synthesis, using L-2-aminobutyrate, which contains an unreactive methyl group (–CH3), instead of a thiol group (–SH). Since ophthalmate lacks a thiol group, it cannot participate in the redox (thiol-mediated) reactions of GSH [10]. The concentration of liver glutathione is the highest in the body, but the liver pool of ophthalmate represents 1% of the size of the glutathione pool, since this compound is excreted to the plasma [10]. Soga et al. recently reported an increase in the concentrations of ophthalmate in mice liver and plasma after induction of oxidative stress [10]. We found a significant increase in plasma and RBCs ophthalmate concentration in animals with chronic hepatitis virus-induced HCC when compared with infected animals without tumors and healthy controls. The function of ophthalmate is still under investigation; Leslie et al. in 2001 showed that ophthalmate could support the uptake of [^3^H]NNAL-O-glucuronide by multidrug resistance protein 1 (MRP1) at levels that were 60% of the GSH-mediated cotransport mechanism, showing that the interaction GSH-MRP1 is not entirely mediated by the thiol group [27]. Ophthalmate was also found to competitively inhibit the canalicular liver plasma membrane GSH transport system [28], resulting in decrease GSH efflux from liver cells during oxidative stress [10]. Since ophthalmate production increase in a state of GSH depletion/oxidative stress, the previously described functions appear to mediate the conservation of intracellular GSH in situations where is most needed. Ophthalmate appears to be a marker of early tumor development and growth in animals with chronic viral hepatitis. 

Factors such as diet, lifestyle, chemical carcinogens and chronic inflammation may result in cellular oxidative stress and it has been shown that reactive oxygen species play a key role in the progression of liver disease. The main sources of hepatic reactive oxygen species are the mitochondria, cytochrome p450, endotoxin-activated macrophages (Kupffer cells), and neutrophils [29], and this has been observed to increase in patients with chronic viral hepatitis, alcoholic hepatitis, and nonalcoholic fatty liver disease [29–31]. Since the glutathione redox state of a patient can change relatively fast, serial plasma measurements of the glutathione system and their analog ophthalmate could prove to be beneficial in the evaluation of patients with liver disease and potentially as a biomarker of HCC.

Previous PET scan imaging studies in our institution have shown that some animals with early-stage HCC (well-differentiated) are not FDG avid, and differences in FDG uptake between HCC and the surrounding hepatic tissues may not be evident. However, both acetate and choline were picked up by this well-differentiated HCC (Figure 1(a)) due to different tracer metabolism between HCC and its surrounding liver tissues [12, 32]. After tumor ablation, lower accessibility of the tracer to the ablated region might contribute to the lower uptake as the microvasculatures might be sealed off by the heat (Figure 1(b)) in addition to the ceased metabolism of necrotic tumor cells as result of the thermal injury. 

Although the WHV model for HCC is a reliable model, it has limitations. Animals develop chronic viral hepatitis resembling the one in the human but limited bridging portal fibrosis with limited portal hypertension. The sample size in the experimental group was limited by the availability of animals, and during the protocol, one animal was excluded due to sickness. Livers had multiple tumors of different sizes, but the correlation of tumor volume with glutathione disturbances and OA concentration was beyond the scope of our present study. Moreover, the redox state of an animal can also be influenced by food intake and genetic factors. In spite of these limitations, our findings resemble those seen in our previous work on the VX2 rabbit model of secondary liver tumors [11] and of others in humans [33].

## 5. Conclusion

Disturbances of the glutathione redox buffer system and higher concentrations of ophthalmate were found in this animal model of woodchuck hepatitis virus-induced HCC. The role of these compounds as biomarkers of early tumor development in patients with end-stage liver disease remains to be determined.

## Abbreviations

HCC: Hepatocellular carcinoma
HBV: Hepatitis B virus
HCV: Hepatitis C virus
WHV: Woodchuck hepatitis virus
GSH: Reduced glutathione
GSSG: Glutathione disulfide
OA: Ophthalmate
RFA: Radiofrequency ablation
RBC: Red blood cell
IAA: Iodoacetate
PET: Positron emission tomography
FDG: 2-deoxy-2[18F]fluoro-D-glucose
LC-MS: Liquid chromatography-mass spectrometry
^3^H: Tritium
NNAL-O-glucuronide: 4-[(methylnitrosamino)-1-(3-pyridyl)-but-1-yl]-b-O-D-glucosiduronic acid
MRP1: Multidrug resistance protein 1
ROS: Reactive oxygen species.
